# Effects of Ubiquinol on Oxidized Low‐Density Lipoprotein in Prediabetic Patients: A Randomized, Double‐Blinded, Placebo‐Controlled Study

**DOI:** 10.1155/bmri/8739655

**Published:** 2026-03-08

**Authors:** Romun Leaovitavat, Pasita Palakornkitti, Paphitchaya Thetsana, Patana Teng-umnuay

**Affiliations:** ^1^ Anti-Aging and Regenerative Medicine, College of Integrative Medicine, Dhurakij Pundit University, Bangkok, Thailand, dpu.ac.th; ^2^ College of Health and Wellness, Dhurakij Pundit University, Bangkok, Thailand, dpu.ac.th

## Abstract

**Background/Objectives:**

Oxidized low‐density lipoprotein (oxLDL) has emerged as a promising biomarker of oxidative burden and cardiovascular risk. This study is aimed at evaluating the effect of ubiquinol supplementation, a potent lipophilic antioxidant, on oxLDL levels in prediabetic patients.

**Methods:**

This was a prospective, randomized, double‐blinded, placebo‐controlled pilot study of 20 adults with impaired fasting plasma glucose (FPG). Participants were randomized to receive either ubiquinol (100 mg/day) or placebo for 12 weeks. Plasma oxLDL levels were measured at baseline and Week 12.

**Results:**

After 12 weeks, the ubiquinol group demonstrated a significant within‐group reduction in plasma oxLDL compared with baseline (*p* = 0.049), whereas no significant change was observed in the placebo group (*p* = 0.237). No adverse events were reported.

**Conclusions:**

Daily ubiquinol (100 mg/day) for 12 weeks was associated with a significant within‐group decrease in plasma oxLDL among prediabetic participants; however, the between‐group difference versus placebo was not significant.

**Trial Registration:**

Thai Clinical Trials Registry: TCTR20250512008

## 1. Introduction

Growing evidence supports the role of oxidized low‐density lipoprotein (oxLDL) in the pathogenesis of atherosclerosis. After low‐density lipoprotein (LDL) accumulates in the subendothelial space of medium‐ to large‐sized arteries, it undergoes lipid peroxidation and phospholipid remodeling (exposure of phosphorylcholine and adduction of aldehyde groups) and becomes oxLDL [1, 2]. oxLDL is primarily taken up by endothelial cells and macrophages leading to endothelial injury, foam–cell formation, and the recruitment of additional inflammatory cells, thereby contributing to the development of atherosclerosis [1, 3, 4].

oxLDL not only accumulates within atherosclerotic plaques but is also detectable in the circulation. Elevated oxLDL levels are strongly associated with increased risk of cardiovascular events, making oxLDL a promising biomarker that more directly reflects oxidative stress and atherosclerotic burden than traditional LDL cholesterol levels [1, 5]. Although antioxidants, including polyphenols, carotenoids, flavonoids, omega‐3 fatty acids, and coenzyme Q10 (CoQ10), have been investigated as potential therapeutic options to limit oxLDL formation, clinical trial evidence for their atheroprotective efficacy remains limited [6–8].

CoQ10 is an essential lipophilic molecule involved in mitochondrial electron transport and adenosine triphosphate production. It exists in two forms: oxidized (ubiquinone) and reduced (ubiquinol). Ubiquinol acts as a powerful antioxidant, capable of directly neutralizing reactive oxygen species (ROS), regenerating other antioxidants (e.g., vitamins C and E), and inhibiting lipid peroxidation [9, 10]. In vitro studies have demonstrated antioxidant effects of CoQ10 against oxidative stress induced by arsenic, zinc, and hydrogen peroxide [11, 12]. CoQ10 also alleviated pancreatic injury in l‐arginine‐induced acute pancreatitis in rat supporting its anti‐inflammatory and antioxidant properties [13]. Wani and Shadab [14] reported the protective effect of CoQ10 against titanium dioxide nanoparticle‐induced oxidative stress in erythrocytes and lymphocytes; oxidative stress was assessed by malonaldehyde and reduced glutathione levels and catalase and superoxide dismutase activities. Compared with ubiquinone, ubiquinol exhibits superior bioavailability and tissue accumulation after oral administration, particularly in metabolically active organs [15].

Through its antioxidant activity, ubiquinol plays a pivotal role in mitigating oxidative stress, a key driver of LDL oxidation and endothelial dysfunction. It scavenges free radicals, stabilizes cellular membranes, preserves mitochondrial integrity, and reduces the formation of oxLDL [16]. In vitro study, Ahmadvand et al. [16] reported a dose‐dependent antioxidant effect of CoQ10 against CuSO_4_‐mediated LDL oxidation, evidenced by reduced formation of conjugated dienes and malonaldehyde and prolongation of the lag time and decrease in electrophoretic mobility. The proposed underlying mechanisms of antioxidant property include the following: (1) independently donating hydrogen atoms to reduce peroxyl and/or alkoxyl radicals and (2) participating in redox interactions with other lipid‐soluble antioxidants, such as vitamin E. Furthermore, studies have shown that CoQ10 supplementation reduces markers of oxidative stress and improves lipid profiles in patients with metabolic disorders, suggesting broader cardioprotective potential [9].

Although the antioxidant effects of ubiquinol have been demonstrated previously, direct evidence of its effect on plasma oxLDL levels or endothelial oxLDL in humans remains limited [9, 16, 17]. Therefore, the present study was designed to investigate the effects of daily ubiquinol supplementation on plasma oxLDL levels in prediabetic patients.

## 2. Materials and Methods

### 2.1. Study Design

A prospective, randomized, double‐blinded, placebo‐controlled pilot study was conducted in 20 Thai adults with prediabetes. The study was approved by the Committee of Human Research Ethics, Dhurakij Pundit University (COA No. 039/66), (TCTR20250512008), and conducted in accordance with the Declaration of Helsinki. Written informed consent was obtained from all participants before study initiation.

### 2.2. Participants

Eligible participants were adults aged 18–60 years with prediabetes, defined as fasting plasma glucose (FPG) between 100 and 125 mg/dL, according to the American Diabetes Association criteria [18], including both newly identified cases at screening and individuals with a prior diagnosis of prediabetes. Eligibility for all participants was confirmed at screening. Exclusion criteria were diagnosis of diabetes mellitus, use of oral hypoglycemic agents, use of vitamins or antioxidant supplements within the past 6 months, uncontrolled hypertension (systemic blood pressure ≥ 160 mmHg or diastolic blood pressure ≥ 100 mmHg), diagnosed or prior history of cardiovascular disease, renal insufficiency, and pregnancy or breastfeeding.

Recruitment was conducted via ethics‐approved research posters distributed on social media platforms and through personal referrals. Eligible participants were enrolled and randomized to receive either ubiquinol (*n* = 10) or placebo (*n* = 10).

For eligibility assessment, approximately 3 mL of venous blood was collected from each participant and analyzed at a standardized laboratory (Alternate Laboratory, Bangkok, Thailand) to determine FPG (milligrams per deciliter) using a chemiluminescent microparticle immunoassay (CMIA). Participants with FPG 100–125 mg/dL who met the inclusion criteria and none of the exclusion criteria were enrolled.

All eligible participants were randomly assigned to either the ubiquinol group (*n* = 10) or the placebo group (*n* = 10) using a computer‐generated block randomization method with a block size of 4. The randomization sequence was concealed in sequentially numbered, opaque, sealed envelopes; allocation was blinded to the enrolling investigator.

Participants in the ubiquinol group received a 500‐mg soft gelatin capsule containing 100 mg of ubiquinol (Kaneka Ubiquinol) emulsified with 400 mg of medium‐chain triglyceride (MCT) oil. According to the certificate of analysis, Kaneka Ubiquinol contains not less than 96.0% ubiquinol and not more than 2.0% ubiquinone. The placebo group received an identical‐appearing soft gelatin capsule containing 500 mg of MCT oil.

All participants were instructed to take one capsule once daily after the first meal of the day for 12 weeks and to maintain their usual diet and lifestyle throughout the study.

### 2.3. Blood Sampling Procedure and Testing

All laboratory procedures were conducted by trained technicians at a private facility (Chemico Asia Health and Beauty Center, Bangkok, Thailand) in accordance with standard operating procedures. Venous blood samples (approximately 10 mL) were collected at baseline and at 12 weeks postintervention. Samples were obtained using vacuum blood collection tubes after a 12‐h overnight fasting and were sent for the determination of the following parameters (Table 1).

**Table 1:** Test parameters, method, unit, and specimen type.

Test parameter	Method	Unit	Specimen type
Fasting plasma glucose (FPG)	CMIA	mg/dL	NaF blood
Creatinine (Cr)	CMIA	mg/dL	Heparinized plasma
Aspartate aminotransferase (AST)	CMIA	U/L	Heparinized plasma
Alanine aminotransferase (ALT)	CMIA	U/L	Heparinized plasma
Complete blood count (CBC)	EI		EDTA
Hemoglobin (Hb)		g/dL	
Hematocrit (Hct)		%	
White blood cell count		Cells/*μ*L	
Platelet		Cells/*μ*L	
Oxidized low‐density lipoprotein (oxLDL)	ELISA	U/L	Heparinized plasma

Abbreviations: CMIA, chemiluminescent microparticle immunoassay; EDTA, ethylenediaminetetraacetic acid; EI, electrical impedance; ELISA, enzyme‐linked immunosorbent assay; NaF, sodium fluoride.

### 2.4. Biochemical Analyses

Biochemical analyses (i.e., FPG, creatinine [Cr], aspartate aminotransferase [AST], and alanine aminotransferase [ALT]) were performed using the Abbott Alinity ci integrated clinical chemistry and immunoassay system (Abbott Laboratories, Chicago, Illinois, United States).

### 2.5. Hematological Analyses

Complete blood count (CBC) was analyzed using the Mindray BC‐5600 automated hematology analyzer (Mindray, Shenzhen, China).

### 2.6. Measurement of oxLDL

Plasma oxLDL was measured using a commercially available oxLDL assay kit (MDA‐LDL, Human; Abcam, Cambridge, United Kingdom) on the BIOBASE1000 automated chemistry analyzer (BIOBASE, Jinan, China). The assay is a competitive enzyme‐linked immunosorbent assay (ELISA) principle, in which malondialdehyde (MDA)‐modified LDL immobilized on the microplate competes with oxLDL present in the sample for binding to a specific monoclonal antibody. Detection is achieved with a horseradish peroxidase (HRP)–conjugated secondary antibody and colorimetric tetramethylbenzidine (TMB) substrate, with absorbance measured at 450 nm. The colorimetric signal is inversely proportional to the oxLDL concentration [19–22].

Briefly, 100 *μ*L of plasma sample was added to an anti‐MDA antibody–coated plate and incubated for 2 h. Following blocking and washing, a biotinylated antihuman Apolipoprotein B‐100 antibody and streptavidin–enzyme conjugate were sequentially applied, followed by TMB substrate. The enzyme reaction was stopped with the stop solution, and absorbance was read immediately at 450 nm using a microplate reader. According to the manufacturer, the assay demonstrates a sensitivity of < 15 ng/mL and a quantifiable range of 0–1 *μ*g/mL.

### 2.7. Clinical and Anthropometric Measurements

Body weight and height were measured using a calibrated digital weighing scale with a stadiometer (Zepper TCS‐250A‐R, Zepper, Thailand). All measurements were recorded to one decimal place. Body mass index (BMI) was calculated using the standard formula: weight (kilograms) divided by height squared (square meters).

Waist circumference was measured using a nonstretchable measuring tape. Measurements were taken at the midpoint between the bottom of the rib and the top of the iliac crest while the participant was standing upright with relaxed arms at the end of a normal expiration, in accordance with World Health Organization guidelines. Two consecutive measurements were taken, and the average value was recorded to the nearest 0.1 cm.

Blood pressure was measured in the seated position after 10 min of rest, using an automated sphygmomanometer (Omron HEM‐7156‐A, Omron Healthcare, Kyoto, Japan). Two measurements were taken at 2‐min intervals, and the average was recorded.

### 2.8. Statistical Analysis

The primary outcome was the difference in plasma oxLDL level between the two groups at 12 weeks. The secondary outcome was the change in plasma oxLDL level at 12 weeks compared to baseline.

Safety was assessed by monitoring clinical symptoms and evaluating laboratory parameters at Week 12 including hemoglobin (Hb), hematocrit (Hct), white blood cell count, platelet, Cr, AST, and ALT.

Frequency and percentage were used to represent categorical variables including sex and underlying disease. Mean and standard deviation (SD) or median and interquartile range (IQR) were used to describe continuous variables depending on the distribution of the data. Normality of continuous variables was assessed using the Shapiro–Wilk test. Continuous variables include age, blood pressure, BMI, waist circumference, FPG, oxLDL, Hb, Hct, white blood cell, platelet, Cr, AST, and ALT levels.

All analyses were performed based on the intention‐to‐treat principle. FPG, oxLDL, Hb, Hct, white blood cell, platelet, Cr, AST, and ALT were compared between the two intervention groups (ubiquinol and placebo) at baseline and Week 12 using either the independent sample *t*‐test or the Wilcoxon rank‐sum test, depending on the distribution of the data. Homogeneity of variance was assessed using Levene′s test when applying independent sample *t*‐tests. The paired sample *t*‐test or Wilcoxon signed‐rank test was applied for comparing pre‐ and postmeasurements within each group. Two‐sided *p* < 0.05 is considered statistically significant. All analyses were performed using Stata statistical software Version 15.0 (StataCorp, College Station, Texas, United States).

## 3. Results

**Figure 1:**
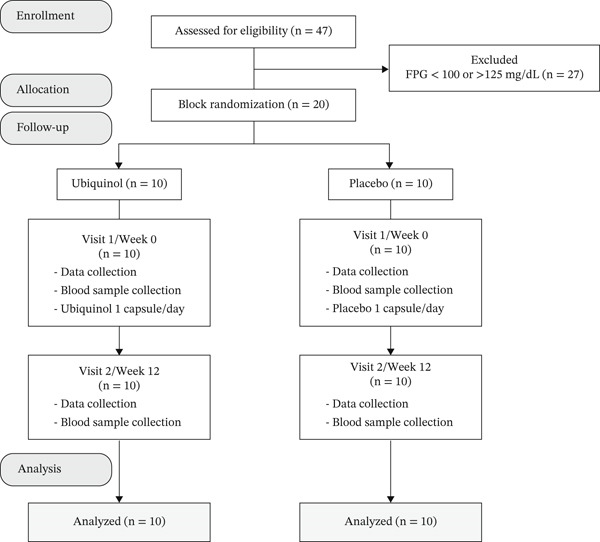
CONSORT diagram of participant flow.

A total of 20 eligible participants were enrolled and completed the 12‐week study. All were included in the final analysis (Figure 1).

At baseline, there were no significant differences between the ubiquinol and placebo groups in clinical and anthropometric parameters (Table 2). The mean ± SD age was 45.4 ± 8.59 years in the ubiquinol group and 47.9 ± 7.50 years in the placebo group (*p* = 0.503). Similarly, sex, underlying diseases, systolic blood pressure, diastolic blood pressure, BMI, and waist circumference were comparable between groups.

**Table 2:** Baseline clinical and anthropometric parameters of participants by treatment group.

	**Placebo group (n = 10) mean ± SD**	**Ubiquinol group(n = 10) mean ± SD**	**p value**
Age (years)	47.9 ± 7.50	45.4 ± 8.59	0.503
Female sex	6 (60%)	7 (70%)	0.639
Underlying disease			
Hypertension	2 (20%)	2 (20%)	0.709
Dyslipidemia	1 (10%)	4 (40%)	0.121
BMI (kg/m^2^)	30.17 ± 5.32	28.68 ± 5.67	0.552
Waist circumference (cm)	97.35 ± 14.28	92.91 ± 14.14	0.494
Systolic blood pressure (mmHg)	126.5 ± 20.84	124.5 ± 19.73	0.828
Diastolic blood pressure (mmHg)	74.6 ± 8.90	81.6 ± 11.44	0.144

Abbreviation: BMI, body mass index.

### 3.1. Biochemical Analyses

Baseline mean ± SD FPG was 107.7 ± 4.60 mg/dL for ubiquinol and 108.6 ± 6.52 mg/dL for placebo. The difference was not significant (*p* = 0.725). No significant between‐group differences in Cr, AST, and ALT were observed at baseline (Table 3). At Week 12, there were no significant between‐group differences of Cr, AST, and ALT (Table 4).

**Table 3:** Baseline biochemical and hematological parameters.

	**Placebo group (n = 10) mean ± SD**	**Ubiquinol group mean ± SD**	**p value**
FPG, mg/dL	108.6 ± 6.52	107.7 ± 4.60	0.725
Cr, mg/dL	0.72 ± 0.19	0.72 ± 0.16	0.980
AST, U/L^a^	27.5 ± 15	25.5 ± 4	0.762
ALT, U/L^a^	33 ± 16	26 ± 23	0.970
Hb, g/dL	13.81 ± 1.36	12.71 ± 1.53	0.106
Hct, %	46.6 ± 4.90	43.5 ± 4.84	0.171
White blood cell count, cells/*μ*L	7307 ± 1986.24	7303 ± 2801.50	0.607
Platelet, ×10^3^ cells/*μ*L	268.4 ± 59.59	277.6 ± 67.28	0.750

Abbreviations: ALT, alanine aminotransferase; AST, aspartate aminotransferase; Cr, creatinine; FPG, fasting plasma glucose; Hb, hemoglobin; Hct, hematocrit.

^a^Data are expressed as median (IQR) due to the nonnormal distribution of data.

**Table 4:** Biochemical and hematological parameters by treatment group at Week 12.

	**Placebo group (n = 10) mean ± SD**	**Ubiquinol group (n = 10) mean ± SD**	**p value**
Cr, mg/dL	0.70 (0.15)	0.71 (0.15)	0.873
AST, U/L^a^	28.5 (27)	24.5 (12)	0.622
ALT, U/L^a^	26 (50)	31.5 (18)	0.970
Hb, g/dL	13.92 (1.39)	12.74 (2.39)	0.192
Hct, %	46.2 (4.34)	43.1 (6.92)	0.246
White blood cell count, cells/*μ*L	7249 (1448.35)	7556 (2083.7)	0.707
Platelet, ×10^3^ cells/*μ*L	277.6 (39.25)	287.6 (71.42)	0.703

Abbreviations: ALT, alanine aminotransferase; AST, aspartate aminotransferase; Cr, creatinine; Hb, hemoglobin; Hct, hematocrit.

^a^Data are expressed as median (IQR) due to the nonnormal distribution of data.

### 3.2. Hematological Analyses

No significant between‐group differences in Hb, Hct, white blood cell count, and platelet count were observed at baseline (Table 3) and Week 12 (Table 4).

### 3.3. Plasma oxLDL

Baseline mean ± SD oxLDL was comparable between the two groups (*p* = 0.645). At 12 weeks, the between‐group difference in oxLDL was not significant (*p* = 0.801). However, the ubiquinol group exhibited a statistically significant within‐group reduction in mean oxLDL from 53.16 ± 6.79 at baseline to 50.16 ± 8.77 after 12 weeks of daily ubiquinol supplement (*p* = 0.049) (Table 5).

**Table 5:** Within‐ and between‐group comparisons of oxLDL from baseline to Week 12.

	**Placebo group (n = 10) mean ± SD**	**Ubiquinol group (n = 10) mean ± SD**	**Between-group** **p value**
oxLDL, U/L			
Baseline	51.65 ± 7.60	53.16 ± 6.79	0.645
Week 12	49.28 ± 8.77	50.16 ± 8.77	0.801
Within‐group *p* value	0.237	0.049^∗^	

Abbreviation: oxLDL, oxidized low‐density lipoprotein.

^∗^Statistical significance of pre‐ and posttreatment difference (within‐group difference) was observed in the ubiquinol group.

### 3.4. Safety Profile

Throughout the 12‐week study, no adverse clinical events such as nausea, vomiting, diarrhea, abdominal pain, or upper respiratory symptoms were reported.

## 4. Discussion

This study evaluated the effects of ubiquinol supplementation on oxLDL levels in prediabetic patients within a randomized, double‐blinded, placebo‐controlled design. Our results demonstrated a significant reduction in oxLDL from baseline within the ubiquinol group after 12 weeks (*p* = 0.049), although the between‐group difference versus placebo at Week 12 was not statistically significant (*p* = 0.801). These findings suggest a potential benefit of ubiquinol in reducing oxidative stress in prediabetic individuals, though further studies with larger sample sizes are needed to confirm the clinical relevance and detect between‐group effects.

oxLDL has emerged as a relevant biomarker for cardiovascular risk due to its direct involvement in atherosclerosis and endothelial dysfunction. Unlike traditional lipid markers such as total cholesterol or LDL, oxLDL reflects oxidative modifications that contribute to plaque formation and vascular inflammation, making it a more specific indicator of cardiovascular disease progression [1, 5]. On this basis, we prespecified oxLDL as the primary biomarker of interest.

Given its superior bioavailability and enhanced tissue distribution, the reduced form of CoQ10, ubiquinol, was selected for this study. Previous pharmacokinetic studies demonstrated that ubiquinol achieves significantly higher plasma concentrations than ubiquinone and is associated with a favorable safety profile even at higher doses [15]. Accordingly, a 100 mg daily dose for 12 weeks was chosen to balance efficacy and safety.

Previous research has demonstrated the benefits of CoQ10 (ubiquinone and ubiquinol) in improving oxidative stress markers, lipid profiles, and endothelial function in various metabolic disorders, including diabetes and cardiovascular diseases [9, 16, 23]. Our study extends these findings to prediabetic patients, who are at heightened risk for cardiovascular complications due to insulin resistance and increased oxidative stress.

Lim et al. [24] observed a significant decrease in plasma ubiquinol/total CoQ10 ratio in subjects with impaired FPG and diabetes compared to healthy subjects. Conversely, the plasma ubiquinone/ubiquinol ratio was significantly higher in subjects with impaired FPG and diabetes compared to healthy subjects. The findings imply that a heightened oxidative burden is present in the stage of impaired FPG or prediabetes.

According to our findings, the reduction in oxLDL within the ubiquinol group can be attributed to several potential mechanisms. First, ubiquinol acts as a lipid‐phase antioxidant by directly donating hydrogen atoms to neutralize lipid peroxyl/alkoxyl radicals, thereby interrupting the propagation phase of lipid peroxidation [9, 16]. Second, ubiquinol regenerates other lipid‐soluble antioxidants, thereby strengthening the endogenous antioxidant system and further preventing oxidation [9, 16]. Third, ubiquinol stabilizes mitochondrial function and lowers mitochondrial ROS generation [9, 10]. Other potential mechanisms include improving lipid metabolism and insulin sensitivity.

Our study did not reach a statistically significant difference in oxLDL levels between the ubiquinol and placebo groups at Week 12. This likely reflects limited statistical power (*n* = 10 per arm) and high variability relative to a modest effect size. Additional contributors may include the ubiquinol dose, 12‐week duration, and the use of a single biomarker.

The within‐group reduction in oxLDL with ubiquinol supplementation aligns with prior reports that antioxidant therapies can improve oxidative biomarkers. Yoo and Yum [17] reported that CoQ10 supplementation significantly improved insulin resistance in prediabetic patients, and Raygan et al. [25] showed the favorable effects of CoQ10 on oxidative stress and lipid profiles in patients with metabolic syndrome.

The safety of ubiquinol supplementation over 12 weeks was also assessed. No clinical side effects such as nausea, vomiting, diarrhea, abdominal pain, or upper respiratory symptoms were reported. Additionally, there were no significant differences between groups in laboratory safety parameters. These findings support short‐term tolerability in prediabetes.

There were some limitations to our study. First, the small sample size may compromise power. Second, the long‐term effect of ubiquinol on oxLDL was not assessed. Third, we measured only a single oxidative biomarker. Further studies with larger sample sizes and longer follow‐up periods are warranted to confirm these findings and to evaluate broader oxidative stress and metabolic endpoints.

## 5. Conclusions

Daily supplementation of ubiquinol (100 mg/day) for 12 weeks was associated with a significant within‐group reduction in plasma oxLDL among prediabetic participants; however, the between‐group difference versus placebo at Week 12 was not significant. These pilot findings warrant confirmation in larger, longer trials.

## Author Contributions

Conceptualization: Romun Leaovitavat and Pasita Palakornkitti; methodology: Romun Leaovitavat and Pasita Palakornkitti; validation: Patana Teng‐umnuay and Paphitchaya Thetsana; formal analysis: Romun Leaovitavat; investigation: Romun Leaovitavat and Pasita Palakornkitti; writing—original draft preparation: Romun Leaovitavat; writing—review and editing: Romun Leaovitavat, Patana Teng‐umnuay, and Paphitchaya Thetsana; supervision: Patana Teng‐umnuay and Paphitchaya Thetsana.

## Funding

No funding was received for this manuscript.

## Disclosure

All authors have read and agreed to the published version of the manuscript.

## Ethics Statement

The study was conducted in accordance with the Declaration of Helsinki and approved by the Human Research Ethics Committee of Dhurakij Pundit University (Protocol Code DPUHREC027/66FB, 29 April 2024).

## Consent

Informed consent was obtained from all subjects involved in the study.

## Conflicts of Interest

The authors declare no conflicts of interest.

## Data Availability

Due to restrictions on data protection, data sharing can only be provided upon request. Requests for access to these materials should be sent via email to the corresponding author of this study.
